# Reproducibility of human landmark identification in morphological mandible prototypes: major parameters for a 3D CBCT approach

**DOI:** 10.1093/fsr/owad029

**Published:** 2023-09-13

**Authors:** Rebeca Menezes Vaz Queiroz Fontes, Tiago Alves de Carvalho Nunes, Ricardo Filipe dos Santos Machado, Patricia Miranda Leite Ribeiro, Jeidson Antônio Morais Marques, Ana Corte-Real

**Affiliations:** Institute of Health Sciences, Federal University of Bahia, Salvador, Bahia, Brazil; Forensic Dentistry Laboratory, Faculty of Medicine, University of Coimbra, Azinhaga de Santa Comba, Celas, Coimbra, Portugal; Forensic Dentistry Laboratory, Faculty of Medicine, University of Coimbra, Azinhaga de Santa Comba, Celas, Coimbra, Portugal; Dentistry School, Federal University of Bahia, Avenida Araújo Pinho, n. 62-Canela, Salvador, Bahia, Brazil; State University of Feira de Santana, Santana, Bahia, Brazil; Institute of Health Sciences, Federal University of Bahia, Salvador, Bahia, Brazil

**Keywords:** forensic sciences, human identification, cone beam computed tomography, anatomic landmarks, mandible

## Abstract

The establishment of anthropometric measurements is of fundamental importance for the correct identification of human bodies. The objective of this study was to evaluate the accuracy and reliability of two-dimensional craniometric landmarks obtained from three-dimensional cone beam computed tomography reconstructions for forensic identification of humans. Computed tomography images with voxel sizes of 0.25, 0.3, and 0.4 mm were obtained using i-CAT® three-dimensional equipment. Ten landmarks were randomly selected, and 10 measurements were demarcated in the three-dimensional reconstruction to evaluate the mandibular condyle, ramus, and body. This study demonstrated that protocols with voxels of 0.3 mm should be preferentially indicated for the evaluation of linear and angular measurements. Implementing our methodology using prototypes for clinical and forensic simulations allows comparisons with human databases in identification issues.

## Introduction

In human identification procedures, bones and teeth are generally used because they are extremely resistant structures of the human body. Despite the various technical possibilities existing in forensic anthropology, the absence of biological characteristics allowing the identification of an individual in cases of air disasters, automobile accidents, homicides, fires, and natural disasters remains a critical problem [1–3]. In many cases, forensic professionals are faced with two major challenges: determination of the cause of death and identification of the person, which includes determining factors such as sex and age [4–7]. Characteristics such as the sex, age, and height of an individual are of great importance in medical-legal practice and can be determined through available methods, especially in cases where a skeletonised body is available for analysis [8].

Several technological resources have considerably improved the techniques and resolution of image capture in the forensic sciences [2, 9, 10], including the facilitation of three-dimensional (3D) image capture techniques. The use of 3D evaluation and interpretation has been shown to be an important tool in forensic analysis. Among the most-used techniques for capturing 3D images is cone beam computer tomography (CBCT), which uses a conical X-ray beam to produce tomographic imaging. The anatomical data acquired can then be manipulated and visualised with specialised software. CBCT technology offers good-quality images with a high resolution and lower cost than conventional computed tomography [11].

The capture of 3D images in the form of successive slices through the body being imaged makes it possible to create 3D models for forensic studies [2, 9, 12, 13]. Thus, it becomes possible to transform images of the human body into anatomical reconstructions that can be used to obtain precise measurements [14]. Rapid prototyping using various processes allows the creation of detailed models with precision that can be evaluated from different perspectives. One of these processes is known as computer-aided design (CAD) [15]. To materialise virtual objects using CAD, a computer-aided manufacturing process was developed that allows a virtual file to be transformed into a real object through 3D printing [16].

The latest innovations in 3D imaging and rapid prototyping procedures are significantly modifying forensic approaches. With technological advances, forensic scientists can reconstruct objects of investigation in 3D [13, 17–19]. Physical human mandible prototypes have been used for various purposes within dentistry, such as testing and improving implants, and maxillofacial surgical planning [20–23]. Thus, they present possibilities for advancing various lines of research within forensic science.

The establishment of anthropometric measurements is of fundamental importance for correct identification of a human body. Correct measurements and interpretations made through physical anthropometry make it possible to define, for example, the dimensions of the human face, with such work using direct measurements that connect defined hard and soft tissue landmarks [6, 24–27]. Several forensic studies [28–31] have used mandibular anthropometric measurements as parameters for postmortem identification. Because of these studies, human landmarks for linear and angular measurements can be precisely located from previously established points described in the literature, and can be standardised for comparison with previous studies [32, 33].

The objective of this study was to evaluate the accuracy and reliability of two-dimensional (2D) craniometric landmarks made from 3D reconstructions created from CBCT data for forensic human identification.

## Materials and methods

The present study describes observational *in vitro* research performed in the Forensic Dentistry Laboratory of the University of Coimbra, Portugal. The research was approved by the Institutional Research Ethics Committee of University of Coimbra (process number CE-112/2019).

The samples used to implement the methodology consisted of 14 randomly selected mandibular resin prototypes, with these simulating several clinical conditions. An alpha value of 5% and beta of 20% were considered for the power calculation, resulting in a sample requirement of 14 specimens considering a test of the difference between two means with dependent groups.

The prototypes were positioned on the equipment and fixed with adhesive tape, using the chin for support and with the mid-sagittal plane perpendicular to the ground, to keep them in a position similar to that in clinical *in situ* imaging. CBCT images were obtained using i-CAT® 3D equipment (i-CAT®, Imaging Sciences International, Hatfield, PA, USA) and were stored in Digital Imaging and Communications in Medicine (DICOM) format with voxel sizes of 0.25, 0.3, and 0.4 mm, exposure time of 0.9 s, and a field of view (FOV) of 100–160 mm. After being recorded and stored in DICOM format to avoid data loss, the images were processed, viewed, manipulated, and analysed in 3D with *in vivo* Dental software version 5.0 (Anatomage, San Jose, CA, USA).

Measurements of the mandible landmarks were made following previously described methods [33]. Three evaluations of each mandible were performed separately by two examiners at three different time points with a minimum interval of 7 days. The examiners, with imaging expertise in the morphometric analysis of CBCT scans, were skilled in the evaluation of mandibular images.

**Figure 1:**
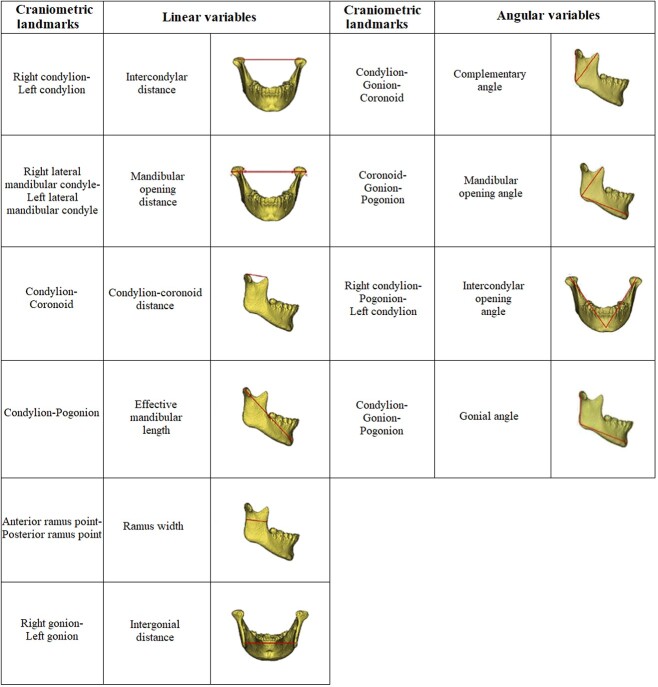
Mandibular measurements and craniometric landmarks.

**Figure 2:**
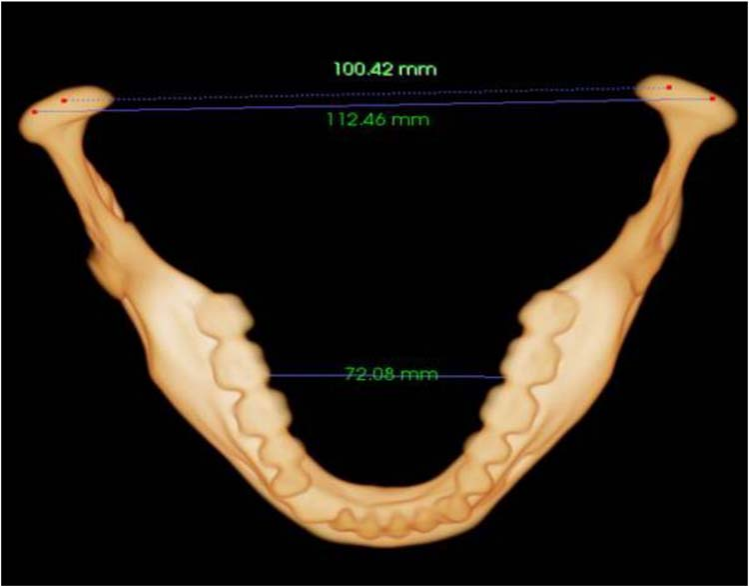
Horizontal mandibular anatomical features (bottom and upper).

Ten landmarks were randomly selected and 10 measures (Figure 1) were demarcated in the 3D reconstruction to evaluate the mandibular condyle, ramus, and body [33]. From these measures, six linear variables and four angular variables were calculated. The selected landmarks and measurements can provide valuable information for mandibular assessment, complementary to or as an alternative to the objectives, in extreme forensic situations [33]. Ten measurements were performed from the landmarks. The specific detection process is shown in Figure 2. At the end of each measurement procedure, the data were exported into Excel (https://www.microsoft.com/en-us/microsoft-365/excel) and saved for subsequent assessment.

Additionally, the measurements obtained from the prototypes were compared with individuals of Portuguese nationality and residency, allowing prediction of age and gender. These data were obtained from a previous study conducted by Coelho et al. [34] with permission.

### Statistical analysis

Intra- and inter-examiner error were calculated using technical error of measurement (TEM) [35]. Data were also analysed with the Bland–Altman method [36] using R version 4.4.2. Intra-examiner error was calculated for each examiner. Inter-examiner error was calculated using the mean value of the three measurements. The Shapiro–Wilk test was used to test the normality of the distribution of measurements [37].

Comparisons between pairs of mean anthropometric measurements and with the data of Coelho et al. [34] were evaluated using Student’s *t*-test for paired samples. The level of significance established for the present study was 0.05.

## Results

### Intra- and inter-examiner analysis

The TEM index allows anthropometrists to verify the degree of accuracy when repeating anthropometrical measurements (intra-examiner) and when comparing their measurements with those of other anthropometrists (inter-examiner) [35]. Comparison of TEM values for intra- and inter-examiner error in relation to linear and angular variables measured from images with different voxel sizes (voxel sizes of 0.25, 0.3, and 0.4 mm) showed acceptable errors in all circumstances [35]. A voxel size of 0.3 mm resulted in the lowest error. Error was observed mainly in intercondylar distance, condylion–coronoid distance, effective mandibular length, ramus width, and intergonial distance (Table 1).

**Table 1:** Technical error measurement (TEM) analysis for intra- and inter-examiner error regarding linear and angular variables.

Variables	Voxel size = 0.25 mm		Voxel size = 0.3 mm		Voxel size = 0.4 mm	
	Intra-examiner TEM (%)	Inter-examiner TEM (%)	Intra-examiner TEM (%)	Inter-examiner TEM (%)	Intra-examiner TEM (%)	Inter-examiner TEM (%)
Linear variables						
Intercondylar distance	0.27	0.50	0.09	0.47	0.44	0.48
Mandibular opening distance	0.24	0.38	0.42	0.61	0.45	1.14
Condylion–coronoid distance	0.28	0.50	0.12	0.46	0.12	0.63
Effective mandibular length	0.10	0.09	0.15	0.01	0.02	0.02
Ramus width	0.25	0.25	0.36	0.21	0.15	0.40
Intergonial distance	0.12	0.35	0.03	0.32	0.09	0.52
Angular variables						
Complementary angle	0.43	0.46	0.25	0.58	0.47	0.49
Mandibular opening angle	0.62	0.07	0.29	0.13	0.21	0.49
Intercondylar opening angle	0.16	0.22	0.22	0.13	0.11	0.51
Gonial angle	0.53	0.26	0.22	0.31	0.20	0.36

The reliability analysis calculated the difference between two measures repeated by the same examiner (intra-examiner), as well as the difference in two measures between different examiners. The values are expressed as mean and standard deviation with their respective confidence intervals. The pairs did not show any statistically significant difference when compared by paired *t*-test. The *P*-values for examiners A (*P* = 0.920) and B (*P* = 0.424) were obtained using paired *t*-tests, and intra-examiner comparisons showed less variability than comparisons made between examiners (Table 2).

**Table 2:** Mean, standard deviation (SD), confidence interval (CI), and *P*-value of 0.3 voxel.

Examiner	Mean	SD	CI (95%)	*P*
Intra-examiner A	0.027	0.830	−0.567–0.621	0.920
Intra-examiner B	0.203	0.766	−0.345–0.751	0.424
Inter-examiner	−0.298	2.096	−1.798–1.202	0.664

**Figure 3:**
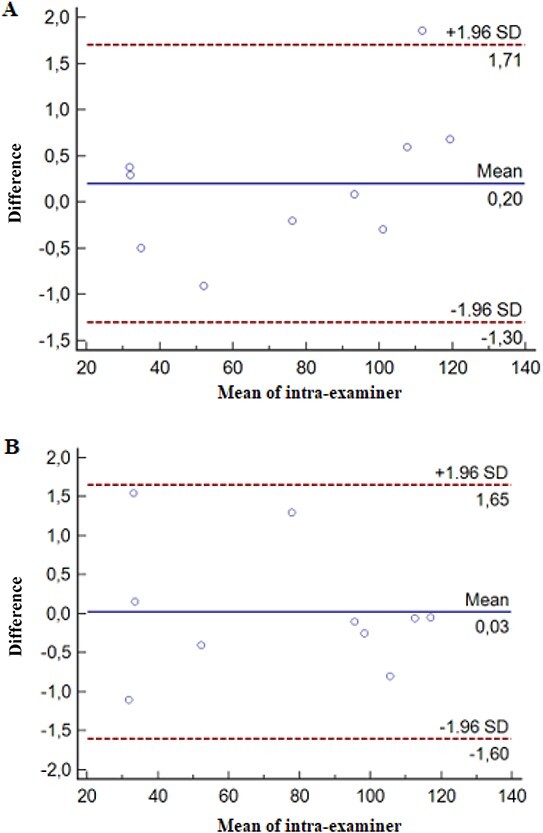
Bland–Altman plot. Mean, standard deviation, confidence interval of examiner A (A) and examiner B (B).

Figure 3 shows a Bland–Altman plot of the differences of two measurements plotted against the average. Horizontal lines are drawn at the mean difference and the limits of agreement defined as the mean difference ± 1.96 SD. It can be concluded that if these limits do not exceed the maximum allowed difference between measurements, the two measurements are considered to be in agreement and may be used interchangeably [36]. According to the Bland–Altman graphs, the mean differences of examiner A and B were 0.2 and 0.03, respectively. It was observed that both examiners had measures close to the mean difference and that remained within the confidence interval.

For the inter-examiner data, Table 2 shows a mean of −0.298, standard deviation of 2.096, confidence interval of −1.798 to 1.202, and *P-*value of 0.664. This demonstrates that although the measures found by the examiners were not the same, they were close and did not exceed the limits used to verify the reliability of the methods.

**Figure 4:**
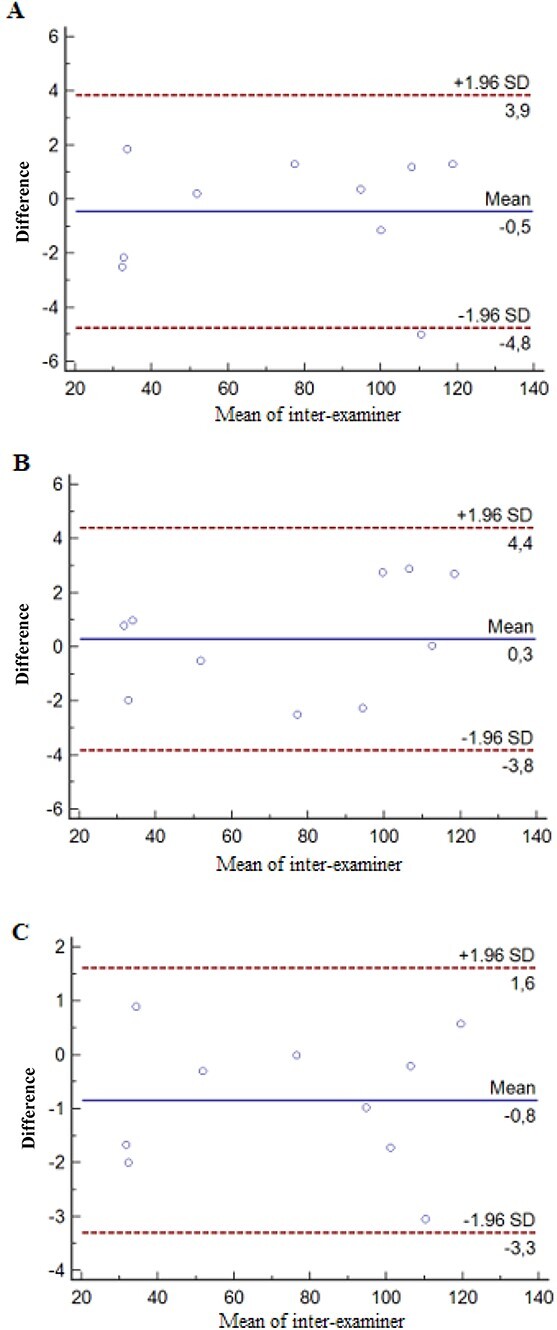
Bland–Altman plots. Inter-examiner data with voxel size 0.25 mm (A), 0.3 mm (B) and 0.4 mm (C).

In the validity investigation, the Bland–Altman plot (Figure 4) confirmed the strong consistency between the examiners, with average differences of −0.5, 0.3, and − 0.8 for voxel sizes of 0.25, 0.3, and 0.4 mm, respectively. It was also observed that the dispersion of the differences plotted against the means varied according to the magnitude of the measurements, and that the error in the measurements of examiners A and B remained stable, regardless of the variables studied.

### Comparison analysis

For the linear and angular variables analysed, the mean values and standard deviation were divided into male and female sex and compared with values for a reference Portuguese population from a previous study [34]. There were no significant differences between the means of the anthropometric measurements, although in both sexes significant differences were found between the mean values of effective mandibular length, intergonial distance, mandibular opening angle, and gonial angle (Table 3).

The *P* values revealed statistically significant differences (*P* < 0.001) in all variables between the female groups in the present study and reference study, while the male groups did not show significant differences in the measurements of intercondylar distance (*P =* 0.068), mandibular opening distance (*P =* 0.121), condylion–coronoid distance (*P =* 0.299), and ramus width (*P =* 0.682) (Table 3).

Low standard deviation was generally observed, indicating that the data points tended to be close to the mean (Table 3).

## Discussion

The use of 3D reproduction technology in forensic sciences was first proposed by Abramov et al. [38] in a study that used laser stereolithography in forensic medicine. Since then, several studies have used 3D printing to provide information for crime solving and to reduce the number of lawsuits filed for lack of evidence [13, 17, 18, 39, 40].

Different methods for capturing images have advantages and disadvantages when compared with each other. The advantages of CBCT over panoramic radiographs are true 3D imaging, no superimposition or distortion, and the ability to create cross-sectional images [41]. The disadvantages over panoramic imaging are increased radiation dose, acquisition artifacts, and cost [41]. Comparisons between CBCT and multislice computed tomography (MDCT) have shown that CBCT has a faster scan time with less potential for movement artifacts, less cost, and less radiation exposure to the patient. A major disadvantage of CBCT is poor soft tissue contrast, which prevents soft tissue assessment [42, 43].

The use of mandible prototypes in dental specialties such as implantology, endodontics, and bucomaxilofacial surgery brings to the forensic sciences the possibility of using these prototypes in simulations of real situations, making study and research in this area of constant development even more viable [20, 22]. Postmortem imaging examinations can facilitate reconstruction of objects targeted for investigation in three dimensions, in addition to having various other utilities, such as evaluating age, sex, and pathology [2, 13]. The present study proposes that the main advantage of working with prototypes in forensic sciences is that it maintains the completeness of the original bone without risking damage to it.

Nica et al. [22] aimed to demonstrate the increased efficiency achieved by dental practitioners when carrying out an *in vitro* training process on a polymeric 3D-printed model before performing *in vivo* surgery. Yoshimura et al. [20] used resin mandible prototypes to assess the outcomes of stereolithographic model-assisted reconstruction of the mandibular condyle with a vascularised fibular flap. The stereolithographic model was used to determine the length and angle of the bony reconstruction.

**Table 3:** Means (M) and standard deviation (SD) regarding linear and angular variables.

	M	M (m)	SD	SD (m)	*P*
Male					
Linear variables					
Intercondylar distance	98.83	97.55	1.06	6.56	0.068
Mandibular opening distance	111.04	109.70	1.37	8.02	0.121
Condylion–coronoid distance	34.97	34.51	1.03	3.54	0.299
Effective mandibular length	119.39	83.35	0.02	8.12	<0.001
Ramus width	31.58	31.44	0.47	3.28	0.682
Intergonial distance	94.42	87.59	0.71	7.57	<0.001
Angular variables					
Complementary angle	33.50	37.36	1.30	4.68	<0.001
Mandibular opening angle	76.60	86.04	0.30	5.97	<0.001
Intercondylar opening angle	52.50	57.46	0.30	4.20	<0.001
Gonial angle	107.50	118.90	0.70	5.94	<0.001
Female					
Linear variables					
Intercondylar distance	98.83	95.58	1.06	6.31	<0.001
Mandibular opening distance	111.04	107.00	1.37	7.00	<0.001
Condylion–coronoid distance	34.97	33.06	1.03	3.41	<0.001
Effective mandibular length	119.39	80.47	0.02	6.21	<0.001
Ramus width	31.58	30.16	0.47	3.40	<0.001
Intergonial distance	94.42	84.88	0.71	5.44	<0.001
Angular variables					
Complementary angle	33.50	36.87	1.30	4.01	<0.001
Mandibular opening angle	76.60	86.21	0.30	6.21	<0.001
Intercondylar opening angle	52.50	58.20	0.30	3.95	<0.001
Gonial angle	107.50	118.70	0.70	5.61	<0.001

M (m/f): mean male or female; SD (m/f): standard deviation male or female; *P* ≤ 0.05.

The software analysis of 3D reconstructions is related to individual performance during anthropometric repetitions. Through such analysis it is possible to examine the variability of measurements due to the diversity of the physical characteristics of the analysed population, biological variation, and technical variations [44].

In this study, we selected linear and angular measurements widely used in 2D analysis in previous studies. The following variables were evaluated in a 3D analysis of the prototypes: intercondylar distance, mandibular opening distance, condylion–coronoid distance, effective mandibular length, ramus width, intergonial distance, complementary angle, mandibular opening angle, intercondylar opening angle, and gonial angle. The craniometric landmarks were all chosen on the basis of previous reference studies [45–48]. The possibility of performing anthropometric measurements from 3D models allows the forensic sciences to evolve technologically, providing reliability and success in the desired interpretations.

The analysis performed in our study showed the variation in the means of each variable between the examiners. Taking into account the margin of error of the method, TEM is usually used as a precision index to represent the quality control of a measure [44]. TEM, which is the standard deviation between repeated measures, is used to calculate intra- and inter-examiner variability [33, 44, 49]. The inter-examiner values are related to non-controllable variables such as the examiner’s skill in identifying the craniometric points. In the present study, they highlight the previous training of the research team. In addition, to verify whether the differences detected in repeated measurements before and after a training session are a result of that training or a result of the relative variation of the method, TEM can be used to estimate whether the confidence intervals around the actual value of the obtained measurement include these variations, thus ensuring the reliability of the measurements performed. Although the findings were not the same, they were close and did not exceed the limits established by the statistical tests used to verify the reliability of the methods.

The way in which the data are collected should be considered when comparing measurements with parameters referred to in the literature because of small variations between values acquired directly and indirectly [50].

The intra- and inter-examiner error was acceptable for all linear and angular variables, which confirms the accuracy of the method reported by Corte-Real et al. [33] and Coelho et al. [34].

The images obtained from the different voxel sizes allowed us to identify all the 10 landmarks and measurements. According to Patcas et al. [51], it is important to compare CBCT examinations with various voxel settings to understand the impact of voxel size on image quality and the reliability and accuracy of diagnostic outcomes. The voxel size may influence noise in the orthogonal sections of an image: the smaller the voxel size, the greater the noise, but also the higher the spatial resolution [52].

In the present study analysing the same measurements for different voxel sizes, we conclude that a voxel size of 0.3 mm is the most appropriate. Table 1 shows that the TEM values of all variables (linear and angular) were lower when a voxel size of 0.3 mm was used, in both intra- and inter-examiner comparisons. Our results also show that volumetric measurements made with CBCT are similar for different voxel sizes, despite a slight tendency toward underestimation, which increased with voxel size. At 0.3 mm and beyond, underestimation of the measurements became statistically significant.

Similar findings were found in a study evaluating the accuracy of software-reformatted panoramic views from CBCT using different voxel sizes [32]. The authors found the smallest error when 0.3-mm voxels were used. According to Torres et al. [53], the four CBCT protocols evaluated, with voxels of 0.2, 0.25, 0.3, and 0.4 mm, were comparable in terms of accuracy of vertical and horizontal measurements, with no significant difference between them. Despite this, they argue that protocols with voxels of 0.3 and 0.4 mm should be preferred in the evaluation of linear measures for the planning of dental implants because of the lower radiation dose.

Corte-Real et al. [33] found that mean values for males were significantly higher than those for females, except for the gonial angle, showing that there are variations in morphology related to sex. These mean values were compared with the results of the present study, and significant differences were found between the mean values measured for both sexes. Comparisons between the measurements of the Portuguese population used in the previous study conducted by Corte-Real et al. [33] and the ones obtained in this present study with 0.3-mm voxels revealed some similar values that match with a male human between the ages of 7 and 15 years. This suggest that 3D physical models can be used to recreate evidence in possible or existing forensic cases, allowing a more detailed analysis without damaging or contaminating the original evidence, thereby revealing the importance of this study.

This study showed that it is possible to create reference points from 3D models, which is an important step for consolidating the use of prototypes. There is a need for more studies to investigate the influence of other factors on human landmarks for linear and angular measurements, besides the influence of voxel size, image quality, and observer performance. Examples of these factors include the selection of reference spots, mouse sensitivity, monitor resolution, and the efficiency of the software used.

## Conclusions

Among the different protocols available for use with CBCT, this study demonstrated that protocols with voxels of 0.3-mm size should be preferentially indicated in the evaluation of linear and angular measurements.

The implementation of prototyping methodology in clinical and forensic simulations allows comparisons with human databases in identification issues, and makes evident the impact of this approach in the forensic area.

The accuracy of the methods used for anthropometric measurements is increasingly becoming consolidated, and this study demonstrated the possibility of identifying 2D craniometric landmarks in 3D reconstructions made from CBCT for human forensic identification.

## Acknowledgements

The authors would like to thank the Laboratory of Forensic Dentistry and the Faculty of Medicine of the University of Coimbra.

## Authors’ contributions

Rebeca Menezes Vaz Queiroz Fontes carried out bibliographic survey of databases, participated in the execution of research in the laboratory, performed data analysis, wrote the article, and adapted the article to the standards of the journal. Tiago Alves de Carvalho Nunes and Ricardo Filipe dos Santos Machado carried out a bibliographic survey of databases, performed the laboratory stage of the research, and participated in the data analysis and writing of the article. Patricia Miranda Leite Ribeiro, Jeidson Antônio Morais Marques, and Ana Corte-Real, as guiding teachers, participated in the design and elaboration of the project, and were present assisting the team in the discussions of data analysis and throughout the process until the submission of the article. All authors contributed to and approved the final text.

## Compliance with ethical standards

The research was approved by the Institutional Research Ethics Committee of University of Coimbra (process number CE-112/2019).

## Disclosure statement

The authors report there are no competing interests to declare.
